# Circulating Bile Acids and Adenoma Recurrence in the Context of Adherence to a High-Fiber, High-Fruit and Vegetable, and Low-Fat Dietary Intervention

**DOI:** 10.14309/ctg.0000000000000533

**Published:** 2022-09-16

**Authors:** Doratha A. Byrd, Maria Gomez, Stephanie Hogue, Gwen Murphy, Joshua N. Sampson, Emily Vogtmann, Paul Albert, Neal D. Freedman, Rashmi Sinha, Erikka Loftfield

**Affiliations:** 1 Division of Cancer Epidemiology and Genetics (DCEG), National Cancer Institute, National Institutes of Health, Rockville, Maryland, USA;; 2 Division of Population Sciences, Department of Cancer Epidemiology, Moffitt Cancer Center, Tampa, Florida, USA;; 3 Department of Surgery and Cancer, Cancer Screening and Prevention Research Group (CSPRG), Imperial College London, London, United Kingdom.

## Abstract

Diet may affect bile acid (BA) metabolism and signaling. In turn, BA concentrations may be associated with cancer risk. We investigated (i) associations of BA concentrations with adenoma recurrence and (ii) the effect of a high-fiber, high-fruit and vegetable, and low-fat dietary intervention on serum BA concentrations.

**METHODS:**

The Polyp Prevention Trial is a 4-year randomized, controlled trial that investigated the effect of a high-fiber, high-fruit and vegetable, and low-fat diet on colorectal adenoma recurrence. Among 170 participants who reported adhering to the intervention and 198 comparable control arm participants, we measured 15 BAs in baseline, year 2, and year 3 serum using targeted, quantitative liquid chromatography-tandem mass spectrometry. We estimated associations of BAs with adenoma recurrence using multivariable logistic regression and the effect of the dietary intervention on BA concentrations using repeated-measures linear mixed-effects models. In a subset (N = 65), we investigated associations of BAs with 16S rRNA gene sequenced rectal tissue microbiome characteristics.

**RESULTS:**

Baseline total BA concentrations were positively associated with adenoma recurrence (odds ratio_Q3 vs Q1_ = 2.17; 95% confidence interval = 1.19–4.04; *P*_trend_ = 0.03). Although we found no effect of the dietary intervention on BA concentrations, pretrial dietary fiber intake was inversely associated with total baseline BAs (Spearman = −0.15; *P*_FDR_ = 0.02). BA concentrations were associated with potential colorectal neoplasm-related microbiome features (lower alpha diversity and higher *Bacteroides* abundance).

**DISCUSSION:**

Baseline circulating BAs were positively associated with adenoma recurrence. Although the dietary intervention did not modify BA concentrations, long-term fiber intake may be associated with lower concentrations of BAs that are associated with higher risk of adenoma recurrence.

## INTRODUCTION

Multiple circulating bile acids (BAs), particularly conjugated BAs, have been associated with higher risk of colorectal cancer (CRC) (1,2), which continues to be the second leading cause of cancer deaths overall in the United States (3). Primary BAs are synthesized from cholesterol in the liver, stored in the gallbladder, and released into the small intestine to aid in the digestion and absorption of fat. Approximately 5% of primary BAs escape enterohepatic circulation, making their way to the colon where they can be transformed to secondary BAs, such as deoxycholic acid, by gut bacteria (4–6). Experimental evidence indicates that secondary BAs are carcinogenic to the colon, and deoxycholic acid causes DNA damage and promotes colon tumor growth (7,8).

Previous literature suggests that BAs may be moderately to strongly influenced by diet. In particular, animal and *in vitro* studies support the possible roles of fat and fiber intake in regulating BA synthesis and excretion, respectively (5,9–11). Few studies have been conducted in humans and most measured fecal BA concentrations, with some finding that intakes of fiber and fat were inversely and positively associated with fecal BA concentrations, respectively (12–21). Previously, we conducted a cross-sectional investigation of the associations of *a priori* selected dietary components with circulating BAs in the prostate, lung, colorectal, and ovarian cancer screening trial (PLCO; US men and women) and the alpha-tocopherol, beta-carotene cancer prevention (ATBC) study (Finnish male smokers) (22). In ATBC, we found that fiber and certain subtypes of fats (i.e., transfat) were inversely and positively associated with multiple BAs, respectively. However, in PLCO, most associations failed to replicate. Therefore, there is a need to better characterize the effects of diet on circulating BAs.

In this study, among men and women with a history of colorectal adenomas, we investigated the associations of circulating BAs with colorectal adenoma recurrence and with the rectal tissue microbiome. We also investigated the effect of adherence to a high-fiber, high-fruit and vegetable, low-fat dietary intervention on circulating BA concentrations and temporal variability of BAs.

## METHODS

### Study design and population

**Figure 1.**
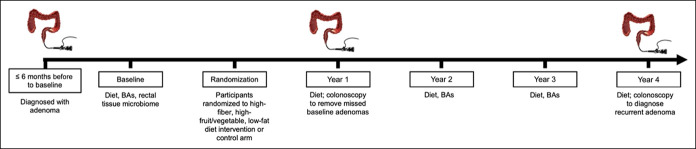
Study design and data included at each time point for the analysis of effects of the high-fiber, high-fruit and vegetable, and low-fat dietary intervention on circulating BAs and of BAs with adenoma recurrence in the Polyp Prevention Trial, 1991–1998. BA, bile acid.

The Polyp Prevention Trial (PPT) (23) is a 4-year multicenter, randomized, controlled trial that investigated the effect of a high-fiber (18 g/1,000 kcal/d), high-fruit and vegetable (3.5 servings/1,000 kcal/d), and low-fat (≤20% of total kcal/d) diet on colorectal adenoma recurrence. The study design is presented in Figure 1. The original study included men and women older than 35 years, with ≥1 histologically confirmed colorectal adenomatous polyp removed 6 months before baseline. Exclusion criteria and changes in dietary intake among the intervention arm are detailed elsewhere (23,24).

### Adenoma assessment

A colonoscopy was conducted 180 days to 2 years after randomization (around year 1) to remove any lesions missed by the baseline colonoscopy. Any colorectal adenoma identified thereafter was considered a recurrent colorectal adenoma. Those without a recurrent adenoma were considered a noncase. For our main analyses, those with hyperplastic polyps were included in the noncase group. For the secondary analyses by adenoma/polyp characteristics, we included hyperplastic polyps as a separate histology, combined advanced (lesions with a maximal diameter of ≥1 cm, ≥25% villous elements, and/or evidence of high-grade dysplasia, including carcinoma) or ≥2 adenomas into 1 category, and categorized only individuals without colorectal adenomas or hyperplastic polyps as noncases.

### Dietary intervention

The specific goals of the dietary intervention included (i) limiting fat intake to 20% of total kcal/d, (ii) consuming ≥ 18 g of fiber per 1,000 kcal/d, and (iii) consuming ≥3.5 servings of fruits and vegetables per 1,000 kcal/d. Dietary goals were calculated based on each participant's total energy intake as determined by the baseline food frequency questionnaire (FFQ), and each participant received a communication with their individual dietary goals and behavior-modification techniques. The intervention group continued to receive dietary counseling by a nutritionist throughout the trial.

At each annual follow-up visit, participants answered questionnaires collecting demographic, behavioral, and medical history information and had dietary goal achievement determined by (i) nutritionist assessment and (ii) their responses to a 4-day food record, followed by the Block/National Cancer Institute FFQ modified to assess more detailed intakes of low-fat and high-fiber foods. Each year the investigators also administered unscheduled 24-hour dietary-recall questionnaires to a newly selected 10% random sample. The FFQ ascertained dietary intakes over the past year and average serving sizes. Compared with the 4-day food record and the 24-hour recall, the FFQ slightly overestimated fat and underestimated fiber, fruit, and vegetable intake (24,25).

We included those who self-reported adhering to the dietary intervention (super compliers) (n = 170) and comparable goal-achieving control arm participants (n = 198), among whom the strongest effects of the dietary intervention on adenoma recurrence were observed (26). Composite indices for dietary goal achievement were calculated by summing intervention goals met over the course of the 4-year trial, totaling 12 goals (3 goals/year × 4 years). Participants in the intervention arm were designated as (i) poor compliers (0–3 goals), (ii) inconsistent compliers (4–8 goals), or (iii) super compliers (9–12 goals). Super compliers completed follow-up and had no missing data on dietary goals. We selected goal-achieving participants from the control arm using methods described by Sansbury et al. (26) and Efron and Feldman (27). Goal-achieving control arm participants were selected such that, in a counterfactual world, had these participants been in the intervention arm, they would have also been super compliers. To do this, we selected control arm participants who completed this study and had no missing data on dietary goals over follow-up. Then, using their annual FFQ, we quantified the number of dietary goals (e.g., consuming <20% of kcal/d from fat) met at each follow-up and ranked them based on the sum of goals met over the 4 years. Approximately, 26% of intervention participants were super compliers; therefore, we similarly selected the top 26% of ranked control arm participants into this study. Overall, goal-achieving control arm participants serve as a more comparable group to super compliers than a random selection of control arm participants.

### BA assessment

Fasted, baseline venous blood samples were collected after study enrollment and at the end of each year of the 4-year trial and stored in a central repository at −80 °C. We measured concentrations of 15 primary and secondary BAs and their conjugates in serum samples from baseline, year 2, and year 3 using the Metabolon fully quantitative liquid chromatography-tandem mass spectrometry platform. At baseline, serum samples were collected after colonoscopy, and at year 2 and year 3, no colonoscopy was conducted. In brief, serum samples were spiked with a solution of corresponding labeled internal standards for each of the BAs and were subjected to protein precipitation with acidified methanol. Samples were then centrifuged, and a portion of the clear supernatant evaporated to dryness in a gentle stream of nitrogen at 40 °C. The dried extract was then reconstituted, and an aliquot injected onto an Agilent 1290/Sciex QTRAP 6500 mass spectrometer liquid chromatography-tandem mass spectrometry system equipped with a C18 reverse-phase high-performance liquid chromatography column with acquisition in negative-ion mode. The peak area of each parent or product ion was then measured against the peak area of the respective internal standard parent or product ion. Quantitation was performed using least squares regression analysis generated from fortified calibration standards prepared immediately before each run.

We included 27 replicates each of 2 pooled, blinded, serum quality control samples randomly distributed throughout each batch, which were used to estimate interbatch coefficients of variation. Thirteen of the 15 BAs were quantifiable in ≥95% of quality control samples. Average interbatch coefficients of variation were ≤20%, except for taurolithocholic acid (26%) and tauroursodeoxycholic acid (45%), which had a high percentage of metabolites below the limit of detection and were excluded from further analyses. Samples from the same participant, collected at different times, were ordered adjacent in the same batch but in a random order.

Values of BA concentrations (ng/mL) below the limit of detection were replaced by half of the lowest detected value for that metabolite. For analyses treating BA concentrations as continuous variables, a log_2_ transformation was used to improve normality. Thus, a 1-unit increase in the log_2_ BA variable can be interpreted as a doubling in the concentration (e.g., if the odds ratio = 3, then a doubling in BA concentration carries a 3-fold odds of disease). We created summary BA measures, including total, primary, and secondary. To do this, we summed the individual BA concentrations and log_2_-transformed the sum. Primary BAs included chenodeoxycholic acid, cholic acid, glycocholic acid, glycochenodeoxycholic acid, taurocholic acid, and taurochenodeoxycholic acid while secondary BAs included deoxycholic acid, lithocholic acid, glycodeoxycholic acid, glycolithocholic acid, and taurodeoxycholic acid.

### Rectal tissue microbiome analysis

Briefly, rectal biopsies from baseline, year 1, and year 4 from 455 participants were lysed using an enzymatic cocktail, homogenized in a Bead Ruptor (Omni International, Kennesaw, GA), and centrifuged. The Animal Tissue DNA Extraction Kit (AutoGen, Holliston, MA) was used for DNA extraction. The V4 region of the 16S rRNA gene was polymerase chain reaction-amplified for 30 cycles, and 2 × 250 bp paired-end sequencing was performed on the Illumina MiSeq v2 using the 500-cycle kit (Illumina, San Diego, CA).

Using the DADA2 pipeline 1.2.1 (28), sequence variant tables and phylogenetic trees were generated based on pair-end sequence reads. After merging and error correction, amplicon sequence variants (ASVs; i.e., 100% operational taxonomic units) were identified. Taxonomy was assigned to the resulting ASVs using the SILVA v123 database, and nonbacterial sequences were removed.

Observed ASVs, Shannon Index, and Faith's phylogenetic diversity were computed using QIIME 1.9.1. Based on rarefaction curves for alpha diversity, we rarefied the alpha and beta diversity metrics to 8,000 reads; this reduced the sample size from 1,059 to 1,030 rectal biopsies. For this study, we selected taxa *a priori* based on prior associations with CRC including *Bacteroides*, *Fusobacterium*, *Porphyromonas*, *Parvimonas*, *Peptostreptococcus*, *Gemella*, *Prevotella*, *Solobacterium*, *Dialister*, and order Clostridiales (29,30); 65 individuals were included for these analyses.

### Statistical analysis

We compared baseline participant characteristics between recurrent adenoma cases and nonrecurrent participants using χ^2^ tests for categorical variables, ANOVA for normally distributed continuous variables, and Kruskal-Wallis tests for non-normally distributed variables. We calculated Pearson correlations between each BA.

To assess BA temporal stability, we calculated intraclass correlation coefficients (ICCs) using linear mixed-effects models with a random effect for subject clustered by center, a fixed effect for year, and a covariate for randomization group. To investigate associations of circulating BA concentrations with adenoma recurrence, we modeled BA concentrations continuously (log_2_-transformed) and as tertiles and used multivariable logistic regression. We repeated the analysis stratified by sex, given prior evidence for sex differences in BA-colorectal neoplasm associations (1,31), and conducted analyses of associations of the BAs with recurrent adenoma/polyp characteristics (advanced/multiple and hyperplastic vs early or left vs right colon). We calculated a *P* value for heterogeneity by characteristics using a case-only multivariable logistic regression analysis with subtype as the dependent variable and the BA and additional covariates as the independent variables.

Mean BA concentrations were calculated for each intervention group for baseline, year 2, and year 3. We then conducted analyses estimating the effect of the dietary intervention on mean year 2 and year 3 concentrations of BAs using repeated-measures linear mixed-effects models. The model included a random effect for the subject, the intercept, indicators for the intervention group and follow-up time (baseline or follow-up), and an intervention × follow-up interaction term. We also explored including covariates (e.g., age, smoking history, sex) in the model that may be associated with systemic BA concentrations based on previous literature but found that their inclusion did not meaningfully change our findings. We repeated the above mixed-effects model analyses stratified by participant characteristics, including age; sex; baseline body mass index (BMI); smoking status; baseline adenoma characteristics; and usual pretrial fat, fiber, and fruit/vegetable intakes. We also conducted analyses stratifying by completion of (i) all-fiber goals at T1, T2, and T3; (ii) all-fat goals at T1, T2, and T3; and (iii) all-fruit and vegetable goals at T1, T2, and T3. To assess whether habitual pretrial diet was associated with baseline BA concentrations, we calculated partial Spearman correlations among pretrial fat, fiber, and fruit and vegetable intakes and BA concentrations. Pretrial dietary intakes were energy-adjusted using the nutrient density method. Finally, we calculated Spearman correlations between circulating BAs and baseline microbiome metrics (alpha diversity and *a priori* selected bacterial taxa) that were previously associated with adenoma recurrence in the PPT study and in prior literature.

We included covariates in the above-described multivariable models based on prior literature and biological plausibility, including age at randomization; sex; intervention arm; BMI; baseline adenoma characteristics; year 1 adenoma characteristics; education; study center; baseline smoking status; family history of CRC; regular nonsteroidal anti-inflammatory drug or aspirin use; and total energy, alcohol, red/processed meat, and fiber intakes. We adjusted for multiple testing with false discovery rate (FDR) correction using the Benjamin-Hochberg method.

Institutional review boards from the National Cancer Institute and all participating centers approved of this study (OH91C0159-B; original clinical trial identifier: NCT00339625).

## RESULTS

Characteristics of the study population, stratified by adenoma recurrence status, are presented in Table 1. On average, recurrent adenoma cases were slightly older; more likely to have 1 or 2 advanced adenomas at baseline; and had slightly higher concentrations of taurochenodeoxycholic acid, taurodeoxycholic acid, and taurolithocholic acid. No other baseline characteristics differed between participants with and without adenoma recurrence. The analytic sample included 150 female and 218 male participants. The primary BAs, cholic acid and chenodeoxycholic acid, were strongly correlated (R = 0.83; *P* < 0.001), as were most glycine-conjugated BAs with their taurine-conjugated counterparts (e.g., R for glycocholic acid and taurocholic acid = 0.89; *P* < 0.001; Supplemental Table 1, Supplementary Digital Content 1, https://links.lww.com/CTG/A877). BAs were moderately stable over the 3 time points. The lowest ICC was for glycolithocholic acid (ICC = 0.43; 95% confidence interval [CI] = 0.37–0.48) while the highest ICC was for taurocholic acid (ICC = 0.60, 95% CI = 0.54–0.65; Table 2).

**Table 1.** Selected baseline participant characteristics and BA concentration measurements of recurrent and nonrecurrent participants in the Polyp Prevention Trial, 1991-1998

Characteristics	Recurrent adenoma cases (n = 129)			Noncases (n = 239)			*P* value^a^
	No.	%	Mean (SD)	No.	%	Mean (SD)	
Sociodemographic							
Sex: male	88	68.20		130	54.40		0.01
Race: White	119	92.20		214	89.50		0.51
Age, yr			64.20 (8.47)			60.01 (9.50)	<0.001
Education level: postgraduate college	35	27.10		74	31.00		0.52
Marital status: married	107	82.90		183	76.60		0.20
Baseline health indicators							
BMI, kg/m^2^			27.49 (3.85)			27.33 (3.89)	0.71
Alcohol consumption, g/d			7.26 (13.29)			5.57 (8.87)	0.15
Smoking status							0.68
Never or former	68	52.71		129	53.97		
Current	61	47.30		110	46.00		
Baseline dietary patterns							
Usual no. of meals							
1 or 2/d	92	71.30		166	69.50		0.80
≥3/d	37	28.70		73	30.50		
Typical no. of meals eaten out							
0 to 1/wk	115	89.10		219	91.60		0.55
≥2/wk	14	10.90		20	8.40		
Caloric intake, kcal/d			1,949.19 (739.99)			1,866.12 (573.50)	0.23
Fat, % of calories			30.47 (7.34)			31.70 (7.13)	0.12
Fiber, g/d			22.61 (8.35)			21.90 (9.20)	0.47
Fruit and vegetable, servings/d			5.28 (2.11)			5.15 (2.20)	0.58
Red and processed meats, g/d			78.00 (51.72)			76.93 (45.68)	0.84
Legumes, g/d			16.10 (18.05)			15.56 (21.02)	0.80
Cruciferous vegs, g/d			29.65 (26.78)			31.14 (22.59)	0.57
Bran cereals, g/d			14.89 (20.80)			14.00 (22.03)	0.71
Total carotenoids, μg/d			10,263.46 (4,910.22)			10,207.61 (5,281.08)	0.92
Baseline vitamin and mineral intake							
Calcium from food, mg/d			941.70 (491.67)			884.92 (409.16)	0.24
Calcium supplement use, mg/d			99.94 (325.60)			175.08 (431.35)	0.09
FFQ: folate, μg/d			367.73 (142.28)			341.12 (133.25)	0.08
FFQ: vitamin E, food, IU			8.57 (3.42)			8.32 (3.02)	0.47
NSAIDs, mg			72.09 (235.65)			120.49 (346.01)	0.16
Multiple vitamin use	51	39.50		98	41.00		0.87
Trial characteristics							
Randomization arm							0.08
Super complier^b^	51	39.50		119	49.80		
Goal achiever^C^	78	60.5		120	50.2		
Days from randomization to T4 visit			1,505.36 (163.28)			1,482.36 (138.42)	0.16
Time from T1 to T4 colonoscopy, d			1,127.74 (177.34)			1,098.46 (174.25)	0.13
No. of trial colonoscopies			1.98 (0.15)			1.96 (0.19)	0.46
Baseline adenoma characteristics							
Size of largest adenoma, cm							1.00
≥ 1	118	91.50		218	91.20		
< 1	1	0.80		2	0.80		
Missing	10	7.80		19	7.90		
Had more than 1 adenoma	4	3.10		1	0.40		0.10
Had a villous/mixed adenoma							0.81
0	104	80.60		196	82.00		
1	23	17.80		41	17.20		
2	2	1.60		2	0.80		
Has an advanced adenoma^d^							<0.001
0	0	0.00		239	100.00		
1	105	81.40		0	0.00		
2	24	18.60		0	0.00		
Had a high-grade adenoma							0.86
0	120	93.00		225	94.10		
1	8	6.20		13	5.40		
2	1	0.80		1	0.40		
Baseline BA concentration, ng/mL							
Chenodeoxycholic acid			156.67 (243.27)			112.14 (255.83)	0.11
Cholic acid			116.03 (245.27)			90.15 (208.70)	0.29
Deoxycholic acid			172.66 (143.91)			157.46 (142.72)	0.33
Glycochenodeoxycholic acid			338.96 (497.93)			250.01 (448.23)	0.08
Glycocholic acid			133.67 (324.54)			102.80 (288.00)	0.35
Glycodeoxycholic acid			193.09 (692.44)			116.06 (158.42)	0.10
Glycolithocholic acid			7.88 (18.95)			6.08 (6.70)	0.19
Glycoursodeoxycholic acid			46.86 (63.33)			35.59 (50.69)	0.06
Lithocholic acid			7.70 (13.36)			6.11 (4.78)	0.10
Taurochenodeoxycholic acid			54.67 (105.28)			33.84 (58.90)	0.02
Taurocholic acid			22.54 (62.50)			15.26 (60.40)	0.28
Taurodeoxycholic acid			30.68 (95.20)			16.84 (22.87)	0.03
Taurolithocholic acid			1.35 (4.03)			0.77 (0.99)	0.04
Tauroursodeoxycholic acid			2.39 (5.10)			1.80 (2.86)	0.15
Ursodeoxycholic acid			25.98 (38.66)			21.03 (31.12)	0.18

BA, bile acid; BMI, body mass index; FFQ, food frequency questionnaire; NSAID, nonsteroidal anti-inflammatory drug.

a *P* values were estimated using ANOVA for normally distributed continuous variables, Kruskal-Wallis tests for non-normally distributed continuous variables, and χ^2^ tests for categorical variables.

b Super compliers were defined as participants in the Polyp Prevention Trial intervention arm who completed all 4 annual FFQs and met a total of 9–12 FFQ goals over the trial period.

c Dietary intervention goals met among the control arm (based on their FFQ) were ranked, and goal-achieving controls were defined as the top 26% of ranked participants.

^d^Advanced adenomas were defined as lesions ≥1 cm in diameter, with at least 25% tubular villous/villous histology or with high-grade dysplasia, including carcinoma.

**Table 2.** Temporal variability of BAs over 3 years in the Polyp Prevention Trial, 1991–1998 (N = 368)

BA	Mean BA concentration, ng/mL			ICC^a^ (95% CI)
	Baseline	Year 2	Year 3	
Chenodeoxycholic acid	127.75	120.25	139.89	0.46 (0.40–0.51)
Cholic acid	99.22	92.10	91.35	0.55 (0.50–0.60)
Deoxycholic acid	162.79	178.83	168.52	0.47 (0.44–0.54)
Glycochenodeoxycholic acid	281.19	250.44	272.38	0.54 (0.48–0.60)
Glycocholic acid	113.62	97.68	110.24	0.55 (0.50–0.60)
Glycodeoxycholic acid	143.06	135.54	143.20	0.49 (0.44–0.54)
Glycolithocholic acid	6.71	6.68	6.88	0.43 (0.37–0.48)
Glycoursodeoxycholic acid	39.54	39.73	44.46	0.54 (0.50–0.58)
Lithocholic acid	6.66	7.67	7.97	0.45 (0.39–0.50)
Taurochenodeoxycholic acid	41.15	35.90	42.97	0.52 (0.47–0.57)
Taurocholic acid	17.81	15.05	23.53	0.60 (0.54–0.65)
Taurodeoxycholic acid	21.69	19.96	22.99	0.50 (0.45–0.55)
Ursodeoxycholic acid	22.77	25.79	29.26	0.48 (0.41–0.54)
Summary scores				
Total bile acids	1,083.96	1,025.62	1,103.66	0.53 (0.46–0.59)
Primary bile acids^b^	226.97	212.35	231.24	0.55 (0.50–0.60)
Secondary bile acids^c^	192.22	212.29	205.75	0.50 (0.44–0.55)

BA, bile acid; CI, confidence interval; ICC, intraclass correlation coefficient.

a Linear mixed-effects models adjusted for the randomization arm were used to calculate ICCs.

b Primary BAs = log_2_ of the sum of chenodeoxycholic acid, cholic acid, glycocholic acid, glycochenodeoxycholic acid, taurocholic acid, and taurochenodeoxycholic acid.

c Secondary BAs = log_2_ of the sum of deoxycholic acid, lithocholic acid, glycodeoxycholic acid, glycolithocholic acid, and taurodeoxycholic acid.

### BA concentrations and adenoma recurrence

Associations of summary BAs at baseline, year 2, and year 3 with adenoma recurrence around year 3–4 of the trial are summarized in Table 3. At baseline, those in the highest relative to lowest tertile of total and primary BAs had a statistically significant 2.17-fold (95% CI = 1.19–4.04; *P*_trend_ = 0.03) and 2-fold (95% CI = 1.10–3.70; *P*_trend_ = 0.03) higher odds of adenoma recurrence, respectively, with associations being stronger among men. Associations of secondary BAs with adenoma recurrence were weaker and not statistically significant. Associations of BAs at year 2 and year 3 with adenoma recurrence were in similar directions but were weaker compared with baseline. At baseline, glycochenodeoxycholic acid and glycocholic acid were most strongly, positively associated with adenoma recurrence, particularly among men (Supplemental Table 2, Supplementary Digital Content 1, https://links.lww.com/CTG/A877). For example, comparing men in the highest relative to lowest tertile of glycochenodeoxycholic acid and glycocholic acid, there were 2.24-fold (95% CI = 0.99–5.20; *P*_trend_ = 0.20) and 2.51-fold (95% CI = 1.13–5.67; *P*_trend_ < 0.001) higher odds of adenoma recurrence, respectively. In our analyses according to adenoma characteristics, baseline BA concentrations were generally inversely associated with hyperplastic polyps and were more strongly, positively associated with adenomas in the left vs right colon (Supplemental Table 3, Supplementary Digital Content 1, https://links.lww.com/CTG/A877).

**Table 3.** Associations^a^ of baseline, year 2, and year 3 BA concentrations with adenoma recurrence in the Polyp Prevention Trial, 1991–1998 (N = 368)

BA concentrations, ng/mL	Overall, N = 368		Female, N = 150		Male, N = 218	
	No.	OR (95% CI)	No.	OR (95% CI)	No.	OR (95% CI)
Baseline						
Total BAs						
Continuous^b^	368	1.27 (1.04–1.56)	150	1.11 (0.77–1.61)	218	1.31 (1.00–1.72)
Tertile 1	123	1.00	57	1.00	66	1.00
Tertile 2	122	1.76 (0.95–3.29)	51	1.38 (0.46–4.19)	71	1.70 (0.75–3.94)
Tertile 3	123	2.17 (1.19–4.04)	42	1.49 (0.46–4.93)	81	2.42 (1.09–5.49)
*P*_trend_^c^		0.03		0.50		0.04
Primary BAs^d^						
Continuous^b^	368	1.20 (1.01–1.42)	150	0.98 (0.72–1.32)	218	1.30 (1.03–1.64)
Tertile 1	123	1.00	62	1.00	61	1.00
Tertile 2	122	1.20 (0.65–2.24)	49	0.78 (0.26–2.27)	73	1.11 (0.46–2.68)
Tertile 3	123	2.00 (1.10–3.70)	39	0.66 (0.19–2.13)	84	2.79 (1.25–6.41)
*P*_trend_^c^		0.03		0.50		0.03
Secondary BAs^e^						
Continuous^b^	368	1.14 (0.97–1.36)	150	1.28 (0.93–1.84)	218	1.07 (0.87–1.33)
Tertile 1	123	1.00	46	1.00	77	1.00
Tertile 2	122	0.99 (0.54–1.80)	57	0.87 (0.29–2.64)	65	1.07 (0.49–2.35)
Tertile 3	123	1.29 (0.72–2.33)	47	1.56 (0.52–4.77)	76	0.99 (0.46–2.10)
*P*_trend_^c^		0.41		0.50		0.99
Year 2						
Total BAs						
Continuous^b^	368	1.15 (0.94–1.41)	150	1.33 (0.92–1.96)	218	0.99 (0.75–1.30)
Tertile 1	123	1.00	61	1.00	62	1.00
Tertile 2	122	1.45 (0.80–2.64)	44	2.68 (0.91–8.24)	78	0.89 (0.41–1.93)
Tertile 3	123	1.18 (0.65–2.16)	45	2.61 (0.76–9.46)	78	0.66 (0.30–1.45)
*P*_trend_^c^		0.85		0.27		0.45
Primary BAs^d^						
Continuous^b^	368	1.15 (0.97–1.37)	150	1.22 (0.89–1.69)	218	1.06 (0.84–1.35)
Tertile 1	123	1.00	64	1.00	59	1.00
Tertile 2	122	2.17 (1.19–3.99)	43	4.37 (1.51–13.61)	79	1.17 (0.53–2.62)
Tertile 3	123	1.41 (0.76–2.62)	43	1.31 (0.34–4.99)	80	1.12 (0.50–2.49)
*P*_trend_^c^		0.84		0.32		0.80
Secondary BAs^e^						
Continuous^b^	368	1.01 (0.87–1.19)	150	1.23 (0.91–1.71)	218	0.88 (0.72–1.08)
Tertile 1	123	1.00	50	1.00	73	1.00
Tertile 2	122	0.77 (0.42–1.39)	56	1.65 (0.56–5.02)	66	0.49 (0.21–1.11)
Tertile 3	123	1.00 (0.56–1.81)	44	2.00 (0.63–6.72)	79	0.60 (0.28–1.27)
*P*_trend_^c^		0.96		0.32		0.45
Year 3						
Total BAs						
Continuous^b^	368	1.09 (0.89–1.32)	150	0.91 (0.65–1.28)	218	1.08 (0.83–1.43)
Tertile 1	123	1.00	60	1.00	63	1.00
Tertile 2	122	1.06 (0.58–1.93)	44	0.49 (0.15–1.50)	78	1.23 (0.55–2.76)
Tertile 3	123	1.40 (0.78–2.52)	46	0.85 (0.30–2.36)	77	1.45 (0.65–3.24)
*P*_trend_^c^		0.38		0.94		0.64
Primary BAs^d^						
Continuous^b^	368	1.08 (0.91–1.28)	150	0.88 (0.65–1.18)	218	1.13 (0.90–1.44)
Tertile 1	123	1.00	63	1.00	60	1.00
Tertile 2	122	0.74 (0.40–1.36)	46	0.77 (0.25–2.27)	76	0.58 (0.25–1.31)
Tertile 3	123	1.51 (0.84–2.73)	41	0.99 (0.34–2.78)	82	1.27 (0.57–2.82)
*P*_trend_^c^		0.38		0.94		0.64
Secondary BAs^e^						
Continuous^b^	368	1.06 (0.90–1.25)	150	1.15 (0.86–1.60)	218	0.93 (0.74–1.17)
Tertile 1	123	1.00	50	1.00	73	1.00
Tertile 2	122	1.44 (0.80–2.61)	48	2.69 (0.93–8.24)	74	0.84 (0.38–1.82)
Tertile 3	123	1.06 (0.59–1.92)	52	0.85 (0.26–2.78)	71	0.84 (0.38–1.82)
*P*_trend_^c^		0.82		0.94		0.65

BA, bile acid; CI, confidence interval; OR, odds ratio.

a Covariates in the multivariable logistic regression models included age at randomization; sex (female or male); intervention arm (control arm or intervention arm); baseline body mass index; baseline adenoma characteristics (advanced/multiple or early adenoma); adenoma characteristics at year 1 (advanced/multiple, hyperplastic, early adenoma, or no polyp); education (college graduate vs postgraduate college); recruitment center (California, New York/Pennsylvania/Illinois/North Carolina/Virginia, Utah); baseline smoking status (current smoker, former smoker, or never regular smoker); family history of colorectal cancer (yes, no, or missing); nonsteroidal anti-inflammatory drug/aspirin use (yes or no); and baseline total energy, alcohol, red/processed meat, and fiber intakes.

b Continuous summary BA scores were log_2_-transformed.

c *P*_trends_ were adjusted for multiple testing using false discovery rate correction using the Benjamin-Hochberg method.

d Primary BAs = log_2_ of the sum of chenodeoxycholic acid, cholic acid, glycocholic acid, glycochenodeoxycholic acid, taurocholic acid, and taurochenodeoxycholic acid.

e Secondary BAs = log_2_ of the sum of deoxycholic acid, lithocholic acid, glycodeoxycholic acid, glycolithocholic acid, and taurodeoxycholic acid.

### Effects of the high-fiber, high-fruit and vegetable, and low-fat dietary intervention

Changes in summary BA concentrations for super compliers relative to goal-achieving control arm participants are summarized in Table 4 (see Supplemental Table 4, Supplementary Digital Content 1, https://links.lww.com/CTG/A877 for changes in individual BA concentrations). We found no appreciable or statistically significant treatment effects of the dietary intervention on BAs, alone or in combination. The effects were similarly null among categories of age; sex; BMI; smoking status; baseline adenoma characteristics; usual pretrial fat/fiber/fruit and vegetable intakes; and adherence to fiber, fat, or fruit and vegetable intervention goals at year 1, year 2, and year 3 (Supplemental Table 5, Supplementary Digital Content 1, https://links.lww.com/CTG/A877).

**Table 4.** Effects of strict adherence to the high-fiber, high-fruit and vegetable, and low-fat diet on summary scores of circulating BA concentrations in the Polyp Prevention Trial, 1991–1998 (N = 170 super compliers and N = 198 goal-achieving controls)

BA concentration, ng/mL	Baseline Mean (95% CI)^a^	Year 2 Mean (95% CI)^a^	Year 3 Mean (95% CI)^a^	Intervention effect, year 2 Beta (SE)^b^	Intervention effect, year 3 Beta (SE)^b^	*P* value^c^
Total bile acids						
Super complier	694.34 (610.27–789.99)	650.34 (571.59–739.90)	690.38 (606.79–785.48)	−0.13 (0.10)	−0.07 (0.10)	0.60
Goal achiever	745.88 (661.81–840.63)	763.36 (677.32–860.33)	778.86 (691.07–877.80)			
Primary bile acids^d^						
Super complier	369.06 (316.30–430.62)	334.52 (286.70–390.30)	356.65 (305.66–416.13)	−0.17 (0.10)	−0.11 (0.10)	0.49
Goal achiever	398.39 (345.32–459.60)	406.76 (352.58–469.26)	416.80 (361.28–480.84)			
Secondary bile acids^e^						
Super complier	194.36 (165.68–228.00)	210.64 (179.56–247.10)	220.16 (187.67–258.27)	0.19 (0.20)	0.21 (0.20)	0.36
Goal achiever	233.85 (201.69–271.13)	222.69 (192.07–258.20)	228.78 (197.32–265.26)			

BA, bile acid; CI, confidence interval.

a Means and 95% CIs are least squared means from linear mixed-effects models, with a random effect for subject and an interaction term for visit × intervention arm.

b Beta coefficients and SEs are from linear mixed-effects models, with a random effect for subject and an interaction term for visit × intervention arm.

c *P* values were estimated using likelihood ratio tests and were adjusted for multiple testing using false discovery rate correction using the Benjamin-Hochberg method.

d Primary BAs = log_2_ of the sum of chenodeoxycholic acid, cholic acid, glycocholic acid, glycochenodeoxycholic acid, taurocholic acid, and taurochenodeoxycholic acid.

e Secondary BAs = log_2_ of the sum of deoxycholic acid, lithocholic acid, glycodeoxycholic acid, glycolithocholic acid, and taurodeoxycholic acid.

To assess whether habitual diet was more strongly associated with BA concentrations, we calculated partial Spearman correlations for associations of baseline fat, fiber, and fruit and vegetable intakes with baseline BA concentrations (Table 5). Fiber was inversely associated with total BAs (R_s_ = −0.15; *P*_FDR_ = 0.02), primary BAs (R_s_ = −0.15; *P*_FDR_ = 0.02), and secondary BAs (R_s_ = −0.14; *P*_FDR_ = 0.03). Of the individual BAs, fiber was most strongly inversely associated with glycochenodeoxycholic acid (R_s_ = −0.17; *P*_FDR_ = 0.07). Fat and fruit/vegetable intakes were not statistically significantly associated with the BAs after FDR correction.

**Table 5.** Spearman correlations^a^ for baseline associations of dietary intervention components with circulating BAs in the Polyp Prevention Trial, 1991–1998 (N = 170 super compliers and N = 198 goal-achieving controls)

BA concentration, ng/mL	Fiber		Fat		Fruits and vegetables	
	R_s_	*P*_FDR_^b^	R_s_	*P*_FDR_^b^	R_s_	*P*_FDR_^b^
Chenodeoxycholic acid	−0.09	0.21	0.00	0.99	0.00	0.99
Cholic acid	−0.04	0.63	−0.02	0.81	0.04	0.65
Deoxycholic acid	−0.10	0.14	−0.04	0.66	0.01	0.90
Glycochenodeoxycholic acid	−0.17	0.07	−0.05	0.60	0.12	0.12
Glycocholic acid	−0.12	0.12	−0.03	0.66	0.12	0.12
Glycodeoxycholic acid	−0.14	0.12	−0.06	0.41	0.10	0.16
Glycolithocholic acid	−0.09	0.18	−0.13	0.12	0.03	0.75
Glycoursodeoxycholic acid	−0.12	0.12	−0.06	0.42	0.11	0.13
Lithocholic acid	−0.09	0.20	−0.11	0.13	0.00	0.99
Taurochenodeoxycholic acid	−0.14	0.12	−0.03	0.66	0.08	0.29
Taurocholic acid	−0.11	0.13	−0.02	0.81	0.09	0.19
Taurodeoxycholic acid	−0.12	0.12	−0.04	0.65	0.10	0.14
Ursodeoxycholic acid	−0.11	0.14	−0.04	0.66	0.06	0.41
Summary scores						
Total bile acids	−0.15	0.02	−0.06	0.38	0.09	0.19
Primary bile acids^c^	−0.15	0.02	−0.03	0.56	0.10	0.12
Secondary bile acids^d^	−0.14	0.03	−0.07	0.25	0.05	0.42

BA, bile acid; FDR, false discovery rate.

a Spearman correlations were adjusted for age at randomization; sex (female/male); intervention arm (control/intervention); body mass index at baseline; baseline adenoma characteristics (advanced/multiple or early adenoma); education (college graduate vs postgraduate college); recruitment center (California, New York/Pennsylvania/Illinois/North Carolina/Virginia, Utah); baseline smoking status (current smoker, former smoker, never regular smoker); family history (yes, no, or missing); regular nonsteroidal anti-inflammatory drug/aspirin use (yes or no); total energy; and nutrient density-adjusted fiber, fat, and fruit and vegetable intakes.

b *P* values adjusted for multiple testing using FDR correction using the Benjamin-Hochberg method.

c Primary BAs = log_2_ of the sum of chenodeoxycholic acid, cholic acid, glycocholic acid, glycochenodeoxycholic acid, taurocholic acid, and taurochenodeoxycholic acid.

d Secondary BAs = log_2_ of the sum of deoxycholic acid, lithocholic acid, glycodeoxycholic acid, glycolithocholic acid, and taurodeoxycholic acid.

### BA concentrations and the rectal microbiome

Among 65 individuals with both baseline BA concentration and *a priori* selected rectal tissue microbiome measurements, multiple BAs were inversely associated with alpha diversity (Table 6) and positively associated with *Bacteroides* relative abundance (Supplemental Table 6, Supplementary Digital Content 1, https://links.lww.com/CTG/A877). For example, the Spearman correlation for the association of secondary BAs with observed ASVs (species richness) was −0.39 (*P* = 0.001) and the Spearman correlation for the association of taurochenodeoxycholic acid with *Bacteroides* abundance was 0.30 (*P* = 0.02).

**Table 6.** Associations^a^ of BA concentrations with rectal tissue microbiome alpha diversity at baseline (N = 65)

BA, ng/mL	Alpha diversity metrics^b^		
	Shannon	Observed ASVs	Faith PD
	Rs; *P*	Rs; *P*	Rs; *P*
Chenodeoxycholic acid	−0.20; 0.12	−0.10; 0.43	−0.08; 0.53
Cholic acid	−0.06; 0.64	0.09; 0.47	0.10; 0.43
Deoxycholic acid	−0.29; 0.02	−0.39; 0.002	−0.22; 0.08
Glycochenodeoxycholic acid	−0.14; 0.28	−0.06; 0.65	−0.20; 0.11
Glycocholic acid	−0.19; 0.13	−0.16; 0.22	−0.25; 0.05
Glycodeoxycholic acid	−0.30; 0.02	−0.33; 0.01	−0.29; 0.02
Glycolithocholic acid	0.02; 0.88	0.07; 0.61	−0.06; 0.67
Glycoursodeoxycholic acid	−0.23; 0.07	−0.30; 0.02	−0.31; 0.01
Lithocholic acid	−0.01; 0.92	−0.001; 0.99	0.06; 0.66
Taurochenodeoxycholic acid	−0.20; 0.12	−0.15; 0.23	−0.28; 0.02
Taurocholic acid	−0.19; 0.13	−0.18; 0.16	−0.31; 0.01
Taurodeoxycholic acid	−0.33; 0.01	−0.39; 0.002	−0.39; 0.002
Ursodeoxycholic acid	−0.24; 0.06	−0.31; 0.01	−0.20; 0.12
Summary scores			
Total BAs	−0.26; 0.04	−0.21; 0.10	−0.26; 0.04
Primary BAs^c^	−0.16; 0.20	−0.03; 0.81	−0.02; 0.90
Secondary BAs^d^	−0.29; 0.02	−0.39; 0.001	−0.24; 0.05

ASV, amplicon sequence variant; BA, bile acid; PD, phylogenetic diversity.

a Spearman correlations were adjusted for age and sex.

b Alpha diversity mean and SD: Shannon Index 5.40 (1.07), observed ASVs 198.68 (67.72), and Faith's PD 21.54 (4.96).

c Primary BAs = log_2_ of the sum of chenodeoxycholic acid, cholic acid, glycocholic acid, glycochenodeoxycholic acid, taurocholic acid, and taurochenodeoxycholic acid.

d Secondary BAs = log_2_ of the sum of deoxycholic acid, lithocholic acid, glycodeoxycholic acid, glycolithocholic acid, and taurodeoxycholic acid.

## DISCUSSION

Among men and women with a history of colorectal adenoma, we found that baseline BA concentrations were positively associated with adenoma recurrence, particularly primary BAs among men. We also found that adhering to a high-fiber, high-fruit and vegetable, and low-fat diet did not alter circulating BA concentrations over 3 years. However, we found that longer term, pretrial fiber intake was inversely associated with BAs at baseline. BA concentrations, predominantly secondary BAs, were inversely associated with rectal microbial alpha diversity and positively associated with abundance of *Bacteroides*, two microbiome metrics that have been associated with colorectal neoplasms generally (29,30) and with adenoma prevalence in PPT (under review).

We found that BAs, particularly glycine-conjugated primary BAs, were positively associated with colorectal adenoma recurrence. Our findings were mostly limited to baseline, possibly reflecting the moderate temporal stability of BAs potentially due to underlying changes in adenoma status across the follow-up period. There is accumulating evidence that dysregulation of BA metabolism may be associated with higher risk of colorectal neoplasms. Human studies have found positive associations of circulating BA concentrations with colorectal adenomas (32–34). A case-control study investigating associations of fecal bacteria with adenoma (n = 233 cases, 547 controls) found that bacterial profiles associated with higher odds of adenoma were consistent with those with the capacity to generate secondary BAs (35). Another small case-control study of 50 adenoma cases and 50 matched controls found that deoxycholic acid was positively associated with adenoma (32). Of note, a limitation of these studies, in contrast to our study, is that they were cross-sectional.

Circulating BAs have also been strongly, positively associated with CRC. In a prospective nested case-control study, those in the highest relative to lowest quartile of glycocholic acid had 2.2-fold higher risk of colon cancer (95% CI = 1.52–3.26). There were similar positive findings for other glycine-conjugated and taurine-conjugated primary and secondary BAs (1). In another prospective nested case-control study in ATBC and PLCO, individual BAs were not associated with CRC risk among men, but among women; 7 primary and secondary BAs were positively associated with risk for incident CRC (e.g., for deoxycholic acid, odds ratio_Q4 v Q1_ = 2.85, 95% CI = 1.45–5.60; *P*_trend_ = 0.011) (2). In contrast to these prior findings, findings for adenoma were strongest among men. The mechanisms underlying potential sex differences in BA-colorectal neoplasm associations requires further investigation but could involve potential BA-hormonal interactions (36).

One mechanism whereby BAs may be associated with higher risk of colorectal neoplasms may be through its bidirectional interactions with the microbiome. Supporting this, we found that secondary BAs were inversely associated with rectal microbiome alpha diversity, which we previously found was cross-sectionally, inversely associated with adenoma prevalence (under review). We also found positive associations of BAs with *Bacteroides*, a bacterium which has been positively associated with adenomas and CRC (30). Given the small number of samples, additional studies of these relationships are needed.

A wealth of evidence from *in vitro* and animal studies demonstrates that dietary fat stimulates hepatocyte secretion of BAs into the bile canaliculi, a necessary function for solubilization and absorption of lipids in the gut (5,9). By contrast, dietary fiber can bind BAs, reducing reabsorption into the terminal ileum and increasing excretion in the stool (10,11). Despite strong biological plausibility, we found that circulating BA concentrations, measured in serum, were not altered by a high-fiber, high-fruit and vegetable, and low-fat dietary intervention. In line with our finding, a dietary intervention study of the effects of flaxseeds (which are high in fiber) on circulating BAs similarly found no intervention effects (37). Furthermore, in a randomized controlled crossover feeding trial of the effects of a whole grain vs refined grain diet (comprising 56 or 25 g fiber/day, respectively) on circulating BAs (N = 80), the whole grain diet paradoxically increased plasma concentrations of taurocholic acid, glycocholic acid, and taurolithocholic acid (38). We previously investigated cross-sectional associations of fat and fiber with circulating BA concentrations in the ATBC and PLCO cohorts (22). In ATBC, we found that transfat and polyunsaturated fat intakes were positively associated with circulating BAs, and monounsaturated fat and fiber intakes were inversely associated with circulating BAs. By contrast, in PLCO, fiber was inversely associated with only tauroursodeoxycholic acid. Taken together, our lack of diet-BA findings could be explained by multiple factors. First, the intervention focused on reducing fat intake as a whole and different subtypes of fats (e.g., trans vs monounsaturated fats) may have opposing effects on BA concentrations, as indicated by our findings in ATBC described above. Second, it is possible that long-term, rather than short-term, dietary patterns may more strongly influence circulating BA concentrations, as suggested by our slightly stronger associations of pretrial fiber and fat intakes with BAs. Third, although we had serial samples collected over 2 follow-up time points, it is possible that, given the moderate BA stability we observed, intraindividual variability attenuated our findings.

Our study had strengths including that it was conducted within a well-characterized, randomized, controlled trial setting with serially collected serum samples. We had detailed diet and lifestyle information and information on adenomas from complete colonoscopies performed at baseline, year 1, and year 4 of the trial. Histologic adenoma characteristics were noted by 2 pathologists independently, decreasing the likelihood of misclassification. Finally, we selected goal-achieving control arm participants as the comparison group for the super compliers in our study, which may be more comparable with super compliers than a random selection of control arm. Study limitations included a relatively healthy, homogenous population with findings that may not apply to the general population. We conducted multiple tests, so chance findings are possible, although we adjusted for multiple testing. We did not have fecal BA data. Serum and fecal BAs are weakly to moderately correlated (39), so it is possible that fecal BAs may be more relevant than circulating BAs for colorectal adenoma risk and effects of diet. For example, in a meta-analysis of the associations between fecal BA concentrations and adenoma risk, total, primary, and secondary BA concentrations were generally, albeit inconsistently, positively associated with risk of adenoma (40). Finally, we leveraged existing microbiome data from rectal tissue, which likely has a different microbiome composition than fecal samples that have been more frequently studied; however, rectal biopsies may be a useful specimen to study the gut microbiome and some research indicates that the microbial communities are relatively homogenous across the colon and rectum (41–43).

In conclusion, we found that circulating BAs, measured at study baseline, were positively prospectively associated with adenoma recurrence over 4 years. However, we did observe that a high-fiber, high-fruit and vegetable, and low-fat diet did not change circulating BA concentrations, at least in the short-term, among individuals with a history of colorectal adenoma. Our findings may facilitate better understanding of the interrelationships among diet, BAs, and colorectal neoplasia. Future investigations with serially collected blood and fecal samples are needed in diverse study populations.

## CONFLICTS OF INTEREST

**Guarantor of the article:** Doratha A. Byrd, PhD, MPH.

**Specific author contributions:** D.A.B., R.S., and E.L. conceptualized the study, analyzed the data, and drafted/edited the manuscript. M.G. and P.A. assisted with statistical analysis planning, data processing and analysis, and manuscript editing. G.M., J.N.S., E.V., and N.D.F. contributed to study design and manuscript editing. S.H. assisted with manuscript editing.

**Financial support:** This study was supported by funding from the Intramural Research Program of the National Cancer Institute at the National Institutes of Health. The content of this publication does not necessarily reflect the views or policies of the Department of Health and Human Services, nor does mention of trade names, commercial products, or organizations implying endorsement by the US Government.

**Potential competing interests:** None to report.

**Patient consent:** Written informed consent was obtained from all participants.

**Data availability:** Data are available upon reasonable request. The microbiome sequencing and corresponding metadata that support the findings of this study are openly available in the National Center for Biotechnology (NCBI) Sequence Read Archive (http://www.ncbi.nlm.nih.gov/bioproject/PRJNA810087; bioproject ID PRJNA810087). This analysis was not preregistered in an independent, institutional registry.

**Study Highlights**

**WHAT IS KNOWN**

✓ Circulating bile acids (BAs) have been associated with higher risk of colorectal cancer.
✓ Few human studies have investigated interrelations of diet, BA concentrations, and colorectal adenoma recurrence.

**WHAT IS NEW HERE**

✓ BA concentrations at baseline were strongly, positively associated with adenoma recurrence.
✓ Although a high-fiber, high-fruit and vegetable, and low-fat diet was not associated with circulating BAs, pretrial fiber intake was inversely associated with total baseline BA concentrations.
✓ BAs may be a potentially intervenable biomarker for colorectal neoplasms.
✓ Additional prospective studies are needed to better elucidate interrelationships among diet, BAs, and colorectal neoplasia.

## Supplementary Material

SUPPLEMENTARY MATERIAL
