# Supplementary material for: Circulating Bile Acids and Adenoma Recurrence in the Context of Adherence to a High-Fiber, High-Fruit and Vegetable, and Low-Fat Dietary Intervention
Source: Clin Transl Gastroenterol. 2022 Sep 16;13(10):e00533. doi: 10.14309/ctg.0000000000000533 (PMC9624497; doi:10.14309/ctg.0000000000000533)
Supplement: SUPPLEMENTARY MATERIAL [file ct9-13-e00533-s001.pdf]

Supplemental Table 1. Pearson correlations[a] for circulating bile acids at baseline in the Polyp Prevention Trial, 1991-1998 (N= 170 super-compliers and N=198 goal-achieving controls)

	CDA	CA	DA	GCDA	GCA	GDA	GLA	GUDA	LA	TCDA	TCA	TDA	UDA	TBA	PBA
CDA															
CA	0.83***														
DA	0.27***	0.23***													
GCDA	0.47***	0.40***	0.11*												
GCA	0.37***	0.36***	0.19***	0.85***											
GDA	0.06	0.09	0.71***	0.53***	0.57***										
GLA	-0.09	-0.04	0.52***	0.24***	0.22***	0.63***									
GUDA	0.48***	0.32***	0.10	0.72***	0.60***	0.33***	0.06								
LA	-0.08	-0.04	0.57***	-0.04	0.03	0.45***	0.69***	-0.13*							
TCDA	0.33***	0.25***	0.01	0.85***	0.81***	0.46***	0.12*	0.58***	-0.06						
TCA	0.23***	0.21***	0.09	0.71***	0.89***	0.49***	0.15**	0.45***	0.01	0.88***					
TDA	-0.03	-0.02	0.53***	0.47***	0.55***	0.84***	0.46***	0.28***	0.32***	0.63***	0.63***				
UDA	0.67***	0.44***	0.28***	0.31***	0.22***	0.05	-0.06	0.68***	-0.08	0.17***	0.10	-0.04			
TBA	0.69***	0.61***	0.45***	0.84***	0.81***	0.57***	0.25***	0.66***	0.11*	0.69***	0.66***	0.47***	0.48***		
PBA	0.74***	0.70***	0.23***	0.88***	0.82***	0.43***	0.15**	0.66***	-0.02	0.76***	0.68***	0.37***	0.45***	0.94***	
SBA	0.18***	0.17***	0.91***	0.36***	0.43***	0.92***	0.64***	0.22***	0.58***	0.28***	0.35***	0.74***	0.16**	0.58***	0.38***

[a] *, **, *** indicate that p-values for spearman correlations were <0.05, <0.01, and <0.001, respectively

Abbreviations: CDA, Chenodeoxycholic acid; CA, Cholic acid; DA, Deoxycholic acid, GCDA, Glycochenodeoxycholic acid; GCA, Glycocholic acid; GDA, Glycodeoxycholic acid; GLA, Glycolithocholic acid; GUDA, Glycoursodeoxycholic acid; LA, Lithocholic acid; TCDA, Taurochenodeoxycholic acid; TCA, Taurocholic acid; TDA, Taurodeoxycholic acid; UDA, Ursodeoxycholic acid; TBA, Total Bile Acids; PBA, Primary Bile Acids; SBA, Secondary Bile Acids.

Supplemental Table 2. Associations[a] of individual bile acid concentrations at baseline with recurrent adenoma in the Polyp Prevention Trial, 1991-1998

		Overall, N= 368	Female, N=150	Male, N=218
**Bile acid concentrations, ng/mL**	**N**	**OR (95% CI)**	**OR (95% CI)**	**OR (95% CI)**
Chenodeoxycholic acid				
Continuous[b]	368	1.07 (0.96, 1.19)	0.96 (0.80, 1.15)	1.12 (0.96, 1.31)
Tertile 1	123	1.00	1.00	1.00
Tertile 2	122	0.89 (0.48, 1.64)	0.82 (0.27, 2.42)	0.72 (0.30, 1.69)
Tertile 3	123	1.27 (0.69, 2.35)	0.48 (0.14, 1.51)	1.42 (0.62, 3.31)
P-trend[c]		0.54	0.63	0.31
Cholic acid				
Continuous[b]	368	1.02 (0.92, 1.12)	0.86 (0.70, 1.03)	1.09 (0.95, 1.24)
Tertile 1	123	1.00	1.00	1.00
Tertile 2	122	0.85 (0.46, 1.54)	0.88 (0.31, 2.45)	0.75 (0.33, 1.69)
Tertile 3	123	1.07 (0.59, 1.94)	0.47 (0.14, 1.49)	1.33 (0.60, 2.92)
P-trend[c]		0.74	0.63	0.43
Deoxycholic acid				
Continuous [b]	368	1.09 (0.93, 1.29)	1.17 (0.86, 1.63)	1.05 (0.86, 1.29)
Tertile 1	123	1.00	1.00	1.00
Tertile 2	122	0.81 (0.44, 1.47)	0.71 (0.24, 2.10)	0.89 (0.41, 1.95)
Tertile 3	123	1.17 (0.65, 2.11)	1.38 (0.48, 4.06)	1.14 (0.53, 2.47)
P-trend[c]		0.69	0.64	0.74
Glycochenodeoxycholic acid				
Continuous[b]	368	1.19 (1.00, 1.42)	1.12 (0.83, 1.52)	1.18 (0.94, 1.50)
Tertile 1	123	1.00	1.00	1.00
Tertile 2	122	1.70 (0.92, 3.18)	2.16 (0.76, 6.35)	1.51 (0.65, 3.61)
Tertile 3	123	2.02 (1.09, 3.81)	1.23 (0.36, 4.00)	2.24 (0.99, 5.20)
P-trend[c]		0.32	0.85	0.20
Glycocholic acid				
Continuous[b]	368	1.18 (1.02, 1.38)	1.11 (0.84, 1.46)	1.23 (1.00, 1.51)
Tertile 1	123	1.00	1.00	1.00
Tertile 2	122	1.09 (0.59, 2.02)	1.26 (0.42, 3.76)	0.83 (0.36, 1.92)
Tertile 3	123	2.09 (1.16, 3.81)	1.18 (0.38, 3.67)	2.51 (1.13, 5.67)
P-trend[c]		0.13	0.85	<0.001
Glycodeoxycholic acid				
Continuous[b]	368	1.13 (0.99, 1.31)	1.29 (0.99, 1.74)	1.07 (0.91, 1.28)
Tertile 1	123	1.00	1.00	1.00
Tertile 2	122	0.93 (0.52, 1.66)	0.94 (0.33, 2.65)	0.86 (0.40, 1.84)
Tertile 3	123	1.07 (0.60, 1.93)	1.70 (0.56, 5.28)	0.82 (0.38, 1.74)
P-trend[c]		0.81	0.63	0.74
Glycolithocholic acid				
Continuous[b]	368	0.95 (0.85, 1.06)	1.02 (0.83, 1.24)	0.95 (0.83, 1.10)
Tertile 1	123	1.00	1.00	1.00
Tertile 2	122	0.71 (0.39, 1.29)	0.32 (0.08, 1.14)	0.85 (0.39, 1.82)

Tertile 3	123	0.93 (0.52, 1.67)	1.07 (0.37, 3.10)	0.80 (0.37, 1.72)
P-trend[c]		0.81	0.63	0.74
Glycoursodeoxycholic acid				
Continuous[b]	368	1.14 (1.01, 1.30)	1.07 (0.86, 1.34)	1.15 (0.97, 1.37)
Tertile 1	123	1.00	1.00	1.00
Tertile 2	122	1.68 (0.93, 3.05)	2.75 (0.93, 8.56)	1.28 (0.59, 2.80)
Tertile 3	123	1.53 (0.84, 2.82)	1.00 (0.32, 3.09)	1.62 (0.74, 3.58)
P-trend[c]		0.54	0.85	0.46
Lithocholic acid				
Continuous[b]	368	0.95 (0.84, 1.08)	0.97 (0.77, 1.26)	0.94 (0.81, 1.10)
Tertile 1	123	1.00	1.00	1.00
Tertile 2	122	0.88 (0.48, 1.62)	0.44 (0.13, 1.39)	1.34 (0.61, 2.98)
Tertile 3	123	0.94 (0.52, 1.68)	1.32 (0.46, 3.89)	0.77 (0.35, 1.67)
P-trend[c]		0.89	0.63	0.53
Taurochenodeoxycholic acid				
Continuous[b]	368	1.16 (1.00, 1.35)	1.13 (0.87, 1.47)	1.13 (0.92, 1.39)
Tertile 1	123	1.00	1.00	1.00
Tertile 2	122	1.62 (0.88, 3.00)	2.02 (0.67, 6.29)	1.33 (0.60, 2.95)
Tertile 3	123	1.62 (0.88, 3.01)	1.79 (0.56, 5.77)	1.54 (0.69, 3.51)
P-trend[c]		0.54	0.64	0.53
Taurocholic acid				
Continuous[b]	368	1.14 (1.00, 1.31)	1.05 (0.84, 1.32)	1.19 (0.99, 1.44)
Tertile 1	123	1.00	1.00	1.00
Tertile 2	122	1.54 (0.84, 2.84)	4.64 (1.54, 15.22)	1.00 (0.44, 2.27)
Tertile 3	123	1.58 (0.87, 2.89)	0.77 (0.21, 2.73)	2.11 (0.96, 4.75)
P-trend[c]		0.54	0.63	0.20
Taurodeoxycholic acid				
Continuous[b]	368	1.09 (0.99, 1.21)	1.20 (0.99, 1.49)	1.04 (0.92, 1.19)
Tertile 1	123	1.00	1.00	1.00
Tertile 2	122	1.05 (0.58, 1.89)	1.42 (0.45, 4.57)	1.03 (0.48, 2.19)
Tertile 3	123	1.25 (0.69, 2.26)	1.76 (0.58, 5.60)	1.05 (0.48, 2.28)
P-trend[c]		0.69	0.63	0.92
Ursodeoxycholic acid				
Continuous[b]	368	1.06 (0.99, 1.14)	0.98 (0.87, 1.10)	1.10 (0.99, 1.22)
Tertile 1	123	1.00	1.00	1.00
Tertile 2	122	1.37 (0.75, 2.52)	1.66 (0.61, 4.68)	0.99 (0.43, 2.25)
Tertile 3	123	1.62 (0.89, 2.98)	0.72 (0.22, 2.31)	1.89 (0.88, 4.17)
P-trend[c]		0.54	0.63	0.20

[a] Covariates in the multivariable logistic regression models included: age at randomization, sex (female or male), intervention arm (control arm or intervention arm), baseline BMI, baseline adenoma characteristics (advanced/multiple or early adenoma), adenoma characteristics at year 1 (advanced/multiple, hyperplastic, early adenoma, or no polyp), education (college graduate vs. post-graduate college), recruitment center (CA, NY/PA/IL/NC/VA, UT), baseline smoking status (current smoker, former smoker, or never regular smoker), family history of colorectal cancer (yes, no, or missing), NSAID/Aspirin use (yes or no), and baseline total energy, alcohol, red/processed meat, and fiber intakes

[b] Continuous bile acids were log2 transformed

[c] P-trends were adjusted for multiple testing using False-Discovery Rate correction using the Benjamin-Hochberg method
Abbreviations: BMI, Body mass Index; CA, California; CI, confidence interval; IL, Illinois; NC, North Carolina; NY, New York; OR, odds ratios; PA, Pennsylvania; UT, Utah; VA, Virginia; NSAID, non-steroidal anti-inflammatory drug

Supplemental Table 3. Associations[a] of bile acid concentrations at baseline with recurrent adenoma/polyp characteristics in the Polyp Prevention Trial, 1991-1998

	Recurrent Polyp Characteristics						Colorectal Site			
	Advanced/Multiple (N=61)		Hyperplastic (N=43)		Early Adenoma (N=68)		Left Colon[b] (N= 43)		Right Colon[c] (N= 67)	
Bile acid concentrations, ng/mL	N	OR (95% CI)	N	OR (95% CI)	N	OR (95% CI)	N	Baseline	N	OR (95% CI)
Chenodeoxycholic acid										
Continuous[d]	129	1.08 (0.93, 1.25)	111	0.87 (0.74, 1.01)	172	1.02 (0.90, 1.16)	301	1.06 (0.91, 1.23)	325	1.02 (0.89, 1.16)
Tertile 1	37	1.00	40	1.00	57	1.00	11	1.00	23	1.00
Tertile 2	39	0.67 (0.35, 1.29)	38	0.66 (0.34, 1.28)	54	0.95 (0.51, 1.78)	17	1.82 (1.13, 2.92)	16	0.46 (0.23, 0.94)
Tertile 3	53	1.14 (0.62, 2.10)	33	0.46 (0.23, 0.94)	61	1.05 (0.56, 1.97)	15	1.52 (0.94, 2.46)	28	0.82 (0.43, 1.57)
P-trend[e]		0.53		0.26		0.87		0.97		0.97
P-hetero		0.19		0.61		Ref		0.76		Ref
Cholic acid										
Continuous[d]	129	1.02 (0.90, 1.16)	111	0.92 (0.80, 1.07)	172	1.00 (0.88, 1.12)	301	0.98 (0.85, 1.13)	325	1.02 (0.90, 1.15)
Tertile 1	38	1.00	40	1.00	58	1.00	14	1.00	20	1.00
Tertile 2	40	0.56 (0.29, 1.09)	36	0.49 (0.24, 0.97)	51	0.93 (0.50, 1.73)	18	1.04 (0.60, 1.80)	16	0.54 (0.27, 1.06)
Tertile 3	51	1.15 (0.63, 2.11)	35	0.71 (0.36, 1.39)	63	0.87 (0.46, 1.66)	11	0.66 (0.38, 1.13)	31	1.02 (0.55, 1.86)
P-trend[d]		0.53		0.9		0.87		0.9		0.90
P-hetero		0.59		0.77		Ref		0.68		Ref
Deoxycholic acid										
Continuous[d]	129	0.99 (0.82, 1.20)	111	0.92 (0.76, 1.12)	172	1.17 (0.96, 1.42)	301	1.29 (1.01, 1.64)	325	1.01 (0.84, 1.21)
Tertile 1	45	1.00	39	1.00	62	1.00	11	1.00	29	1.00
Tertile 2	35	0.80 (0.42, 1.53)	35	0.98 (0.52, 1.84)	53	0.82 (0.43, 1.57)	14	1.36 (0.85, 2.16)	15	0.53 (0.26, 1.06)
Tertile 3	49	0.75 (0.40, 1.42)	37	0.41 (0.21, 0.83)	57	1.30 (0.72, 2.36)	18	1.75 (1.08, 2.83)	23	0.85 (0.44, 1.63)
P-trend[e]		0.56		0.15		0.85		0.90		0.97
P-hetero		0.24		0.12		Ref		0.13		Ref
Glycochenodeoxycholic acid										
Continuous[d]	129	1.08 (0.87, 1.34)	111	0.67 (0.52, 0.86)	172	1.15 (0.94, 1.41)	301	1.25 (0.99, 1.56)	325	1.08 (0.88, 1.32)
Tertile 1	31	1.00	37	1.00	53	1.00	11	1.00	17	1.00
Tertile 2	42	1.26 (0.66, 2.40)	35	0.48 (0.24, 0.96)	55	1.75 (0.90, 3.39)	11	1.27 (0.75, 2.16)	23	1.51 (0.75, 3.05)
Tertile 3	56	1.24 (0.67, 2.31)	39	0.34 (0.16, 0.69)	64	2.18 (1.17, 4.08)	21	2.35 (1.40, 3.93)	27	1.49 (0.74, 3.01)
P-trend[e]		0.70		0.10		0.52		0.90		0.90
P-hetero[e]		0.87		0.03		Ref		0.29		Ref
Glycocholic acid										
Continuous[d]	129	1.06 (0.87, 1.29)	111	0.68 (0.53, 0.88)	172	1.17 (0.98, 1.39)	301	1.17 (0.96, 1.43)	325	1.06 (0.89, 1.28)
Tertile 1	34	1.00	37	1.00	57	1.00	11	1.00	20	1.00
Tertile 2	37	0.52 (0.26, 1.07)	37	0.43 (0.22, 0.86)	50	1.50 (0.77, 2.90)	15	1.42 (0.87, 2.32)	18	0.88 (0.43, 1.77)
Tertile 3	58	1.20 (0.65, 2.25)	37	0.29 (0.14, 0.61)	65	2.31 (1.22, 4.37)	17	1.77 (1.09, 2.87)	29	1.43 (0.74, 2.76)
P-trend[e]		0.53		0.10		0.52		0.90		0.90
P-hetero		0.89		<0.001		Ref		0.53		Ref
Glycodeoxycholic acid										
Continuous[d]	129	1.01 (0.85, 1.19)	111	0.87 (0.75, 1.01)	172	1.22 (1.03, 1.45)	301	1.33 (1.08, 1.62)	325	1.05 (0.89, 1.23)
Tertile 1	43	1.00	37	1.00	62	1.00	9	1.00	26	1.00
Tertile 2	40	0.66 (0.34, 1.25)	37	0.82 (0.43, 1.58)	55	1.17 (0.62, 2.20)	18	2.20 (1.40, 3.46)	20	0.87 (0.44, 1.71)
Tertile 3	46	0.53 (0.28, 1.02)	37	0.34 (0.17, 0.68)	55	1.38 (0.74, 2.57)	16	1.77 (1.14, 2.75)	21	0.84 (0.42, 1.66)
P-trend[e]		0.53		0.10		0.85		0.90		0.97
P-hetero		0.18		0.01		Ref		0.05		Ref
Glycolithocholic acid										
Continuous[d]	129	0.88 (0.77, 1.01)	111	0.90 (0.78, 1.05)	172	0.99 (0.86, 1.14)	301	1.09 (0.92, 1.31)	325	0.94 (0.83, 1.06)
Tertile 1	48	1.00	42	1.00	66	1.00	15	1.00	24	1.00
Tertile 2	36	0.59 (0.31, 1.14)	37	0.71 (0.37, 1.34)	52	0.71 (0.38, 1.32)	8	0.51 (0.30, 0.87)	23	1.06 (0.56, 1.98)
Tertile 3	45	0.76 (0.41, 1.41)	32	0.38 (0.19, 0.75)	54	0.82 (0.45, 1.50)	20	1.36 (0.77, 2.40)	20	0.88 (0.45, 1.70)
P-trend[e]		0.53		0.10		0.85		0.90		0.97
P-hetero		0.13		0.3		Ref		0.14		Ref
Glycoursodeoxycholic acid										

Continuous[d]	129	1.09 (0.92, 1.29)	111	0.76 (0.64, 0.91)	172	1.10 (0.93, 1.29)	301	1.20 (1.00, 1.45)	325	1.02 (0.87, 1.19)
Tertile 1	35	1.00	39	1.00	57	1.00	8	1.00	23	1.00
Tertile 2	46	1.01 (0.52, 1.96)	40	0.43 (0.22, 0.87)	58	1.86 (1.00, 3.47)	20	3.38 (2.13, 5.37)	22	1.15 (0.58, 2.29)
Tertile 3	48	1.26 (0.67, 2.38)	32	0.35 (0.17, 0.71)	57	1.28 (0.68, 2.41)	15	2.09 (1.35, 3.24)	22	0.85 (0.42, 1.71)
P-trend[e]		0.56		0.10		0.87		0.90		0.97
P-hetero		0.54		0.03		Ref		0.12		Ref
Lithocholic acid										
Continuous[d]	129	0.91 (0.78, 1.05)	111	1.01 (0.84, 1.22)	172	1.01 (0.86, 1.19)	301	0.98 (0.82, 1.17)	325	0.98 (0.84, 1.14)
Tertile 1	45	1.00	33	1.00	59	1.00	16	1.00	21	1.00
Tertile 2	39	0.69 (0.36, 1.34)	40	1.38 (0.75, 2.53)	55	1.23 (0.67, 2.25)	11	0.72 (0.40, 1.28)	24	1.16 (0.62, 2.18)
Tertile 3	45	0.70 (0.37, 1.31)	38	1.01 (0.53, 1.93)	58	1.24 (0.67, 2.28)	16	0.95 (0.53, 1.70)	22	0.95 (0.50, 1.80)
P-trend[e]		0.53		0.90		0.85		1.00		0.97
P-hetero		0.29		0.60		Ref		0.75		Ref
Taurochenodeoxycholic acid										
Continuous[d]	129	1.13 (0.93, 1.38)	111	0.82 (0.66, 1.03)	172	1.11 (0.93, 1.33)	301	1.12 (0.91, 1.37)	325	1.13 (0.94, 1.35)
Tertile 1	33	1.00	35	1.00	53	1.00	13	1.00	15	1.00
Tertile 2	46	0.91 (0.47, 1.77)	40	0.54 (0.27, 1.08)	57	2.13 (1.16, 3.94)	15	1.19 (0.68, 2.10)	25	2.01 (1.05, 3.86)
Tertile 3	50	1.32 (0.71, 2.46)	36	0.69 (0.35, 1.39)	62	1.65 (0.87, 3.11)	15	1.14 (0.65, 1.99)	27	1.69 (0.88, 3.25)
P-trend[e]		0.53		0.70		0.85		0.97		0.90
P-hetero		0.42		0.12		Ref		0.91		Ref
Taurocholic acid										
Continuous[d]	129	1.09 (0.92, 1.31)	111	0.86 (0.70, 1.06)	172	1.13 (0.96, 1.32)	301	1.06 (0.89, 1.27)	325	1.10 (0.93, 1.29)
Tertile 1	35	1.00	37	1.00	58	1.00	14	1.00	17	1.00
Tertile 2	45	0.57 (0.29, 1.12)	39	0.31 (0.16, 0.60)	54	2.42 (1.33, 4.41)	15	1.40 (0.79, 2.46)	26	1.84 (0.98, 3.45)
Tertile 3	49	1.09 (0.59, 2.02)	35	0.47 (0.24, 0.93)	60	1.68 (0.91, 3.09)	14	0.94 (0.54, 1.66)	24	1.26 (0.67, 2.38)
P-trend[e]		0.53		0.36		0.85		0.97		1.00
P-hetero		0.83		0.03		Ref		0.65		Ref
Taurodeoxycholic acid										
Continuous[d]	129	1.05 (0.92, 1.19)	111	0.94 (0.83, 1.07)	172	1.11 (0.98, 1.26)	301	1.15 (0.99, 1.35)	325	1.07 (0.95, 1.21)
Tertile 1	42	1.00	38	1.00	61	1.00	12	1.00	22	1.00
Tertile 2	40	0.90 (0.47, 1.71)	33	0.62 (0.32, 1.19)	54	1.07 (0.55, 2.06)	13	1.22 (0.71, 2.08)	23	1.27 (0.66, 2.42)
Tertile 3	47	0.71 (0.36, 1.39)	40	0.45 (0.23, 0.86)	57	1.51 (0.82, 2.81)	18	1.50 (0.88, 2.55)	22	1.07 (0.55, 2.09)
P-trend[e]		0.53		0.20		0.85		0.90		1.00
P-hetero		0.73		0.12		Ref		0.57		Ref
Ursodeoxycholic acid										
Continuous[d]	129	1.10 (0.99, 1.23)	111	0.92 (0.84, 1.01)	172	1.00 (0.92, 1.10)	301	1.09 (0.98, 1.22)	325	0.98 (0.90, 1.07)
Tertile 1	35	1.00	41	1.00	54	1.00	11	1.00	23	1.00
Tertile 2	45	1.79 (0.98, 3.28)	35	0.70 (0.35, 1.42)	59	0.97 (0.51, 1.85)	16	1.60 (0.98, 2.60)	21	0.76 (0.38, 1.51)
Tertile 3	49	1.88 (1.04, 3.40)	35	0.62 (0.30, 1.29)	59	1.24 (0.67, 2.30)	16	1.68 (1.04, 2.73)	23	0.96 (0.49, 1.86)
P-trend[e]		0.53		0.49		0.85		0.90		0.97
P-hetero		0.11		0.49		Ref		0.07		Ref
Summary scores										
Total Bile Acids										
Continuous[d]	129	1.11 (0.88, 1.40)	111	0.62 (0.48, 0.81)	172	1.26 (1.01, 1.56)	301	1.36 (1.07, 1.74)	325	1.08 (0.86, 1.35)
Tertile 1	31	1.00	35	1.00	52	1.00	8	1.00	19	1.00
Tertile 2	43	1.08 (0.56, 2.08)	39	0.56 (0.28, 1.12)	56	2.15 (1.15, 4.02)	16	2.31 (1.42, 3.75)	22	1.37 (0.69, 2.72)
Tertile 3	55	1.53 (0.81, 2.87)	37	0.45 (0.22, 0.92)	64	2.29 (1.21, 4.33)	19	2.84 (1.70, 4.74)	26	1.42 (0.71, 2.84)
P-trend[e]		0.21		0.07		0.07		0.04		0.31
P-hetero		0.95		0.02		Ref		0.21		Ref
Primary Bile Acids[f]										
Continuous[d]	129	1.11 (0.90, 1.37)	111	0.66 (0.52, 0.84)	172	1.14 (0.94, 1.39)	301	1.21 (0.98, 1.51)	325	1.09 (0.90, 1.33)
Tertile 1	39	1.00	36	1.00	53	1.00	10	1.00	17	1.00
Tertile 2	38	0.78 (0.40, 1.55)	39	0.70 (0.36, 1.36)	55	1.48 (0.77, 2.82)	13	1.33 (0.81, 2.19)	23	1.22 (0.60, 2.44)
Tertile 3	52	1.42 (0.76, 2.66)	36	0.40 (0.19, 0.85)	64	2.06 (1.09, 3.89)	20	2.46 (1.49, 4.07)	27	1.49 (0.74, 2.97)
P-trend[e]		0.25		0.07		0.07		0.04		0.31
P-hetero		0.60		0.02		Ref		0.47		Ref
Secondary Bile Acids[g]										
Continuous[d]	129	0.99 (0.82, 1.20)	111	0.87 (0.72, 1.04)	172	1.26 (1.03, 1.54)	301	1.44 (1.13, 1.83)	325	1.03 (0.86, 1.24)

Tertile 1	41	1.00	36	1.00	61	1.00	8	1.00	28	1.00
Tertile 2	40	0.77 (0.40, 1.47)	35	0.81 (0.42, 1.56)	52	1.19 (0.63, 2.25)	16	1.96 (1.26, 3.04)	16	0.65 (0.33, 1.32)
Tertile 3	48	0.64 (0.33, 1.23)	40	0.42 (0.20, 0.85)	59	1.82 (0.99, 3.35)	19	2.45 (1.55, 3.86)	23	0.87 (0.45, 1.68)
P-trend[e]		0.21		0.07		0.09		0.04		0.57
P-hetero		0.15		0.03		Ref		0.04		Ref

[a] Covariates in the multivariable logistic regression models included: age at randomization, sex (female or male), intervention arm (control arm or intervention arm), baseline BMI, baseline adenoma characteristics (advanced/multiple or early adenoma), adenoma characteristics at year 1 (advanced/multiple, hyperplastic, early adenoma, or no polyp), education (college graduate vs. post-graduate college), recruitment center (CA, NY/PA/IL/NC/VA, UT), baseline smoking status (current smoker, former smoker, or never regular smoker), family history of colorectal cancer (yes, no, or missing), NSAID/Aspirin use (yes or no), and baseline total energy, alcohol, red/processed meat, and fiber intakes

[b] Left (distal) colon: the largest adenoma was in the splenic flexure, descending, sigmoid, rectum, or rectosigmoid colon.

[c] Right (proximal) colon: the largest adenoma was in the cecum, ascending, hepatic flexure, or transverse colon.

[d] Bile acid concentrations were log2 transformed.

[e] P-trends were adjusted for multiple testing using False-Discovery Rate correction using the Benjamin-Hochberg method.

[f] Primary Bile Acids = log2 of the sum chenodeoxycholic acid, cholic acid, glycocholic acid, glycochenodeoxycholic acid, taurocholic acid, and taurochenodeoxycholic acid

[g] Secondary Bile Acids =log2 of the sum of deoxycholic acid, lithocholic acid, glycodeoxycholic acid, glycolithocholic acid, and taurodeoxycholic acid.

Abbreviations: BMI, Body mass Index; CA, California; CI, confidence interval; IL, Illinois; NC, North Carolina; NY, New York; OR, odds ratios; PA, Pennsylvania; UT, Utah; VA, Virginia; NSAID, non-steroidal anti-inflammatory drug

Supplemental Table 4. Effects of strict adherence to the high-fiber, high-fruit and-vegetable, and low-fat diet on circulating bile acid concentrations in the Polyp Prevention Trial, 1991-1998 (N=170 super-compliers and N=198 goal-achieving controls)

	Baseline	Year-2	Year3	Intervention effect, year-2	Intervention effect, year-3	
**Bile acid concentration, ng/mL**	**Mean (95% CI)[a]**	**Mean (95% CI)[a]**	**Mean (95% CI)[a]**	**Beta (SE)[b]**	**Beta (SE)[b]**	***P*[c]**
Chenodeoxycholic acid						
Super-complier	41.60 (32.80, 52.70)	37.64 (29.7, 47.7)	41.45 (32.7, 52.5)	-0.22 (0.2)	-0.14 (0.2)	0.97
Goal-achiever	40.21 (32.30, 50.10)	42.31 (34.0, 52.7)	44.06 (35.4, 54.9)			
Cholic acid						
Super-complier	23.72 (18.5, 30.5)	21.40 (16.7, 27.5)	20.28 (15.8, 26.0)	-0.26 (0.2)	-0.27 (0.2)	0.87
Goal-achiever	22.90 (18.2, 28.9)	24.74 (19.6, 31.2)	23.66 (18.8, 29.8)			
Deoxycholic acid						
Super-complier	101.33 (85.8, 119.6)	107.46 (91.0, 126.8)	112.03 (94.9, 132.2)	0.16 (0.2)	0.18 (0.2)	0.87
Goal-achiever	112.48 (96.5, 131.2)	107.02 (91.8, 124.8)	109.52 (93.9, 127.7)			
Glycochenodeoxycholic acid						
Super-complier	154.36 (132.6, 179.7)	142.55 (122.4, 166.0)	151.22 (129.9, 176.1)	-0.07 (0.1)	-0.05 (0.1)	0.99
Goal-achiever	168.84 (146.6, 194.4)	163.32 (141.8, 188.1)	170.91 (148.4, 196.8)			
Glycocholic acid						
Super-complier	46.86 (39.6, 55.5)	46.30 (39.1, 54.9)	48.58 (41.0, 57.6)	0.01 (0.2)	-0.02 (0.2)	0.99
Goal-achiever	53.21 (45.5, 62.3)	52.35 (44.7, 61.2)	55.82 (47.7, 65.3)			
Glycodeoxycholic acid						
Super-complier	56.60 (46.5, 68.9)	64.87 (53.3, 78.9)	67.24 (55.2, 81.8)	0.31 (0.2)	0.32 (0.2)	0.87
Goal-achiever	74.31 (61.9, 89.1)	68.93 (57.5, 82.7)	70.70 (58.9, 84.8)			
Glycolithocholic acid						
Super-complier	2.61 (2.1, 3.3)	3.02 (2.4, 3.8)	3.32 (2.6, 4.2)	0.43 (0.3)	0.62 (0.3)	0.59
Goal-achiever	3.79 (3.0, 4.7)	3.25 (2.6, 4.0)	3.14 (2.5, 3.9)			
Glycoursodeoxycholic acid						
Super-complier	19.94 (16.0, 24.8)	16.23 (13.1, 20.2)	17.86 (14.4, 22.2)	-0.29 (0.2)	-0.24 (0.2)	0.87
Goal-achiever	16.67 (13.6, 20.4)	16.54 (13.5, 20.2)	17.63 (14.4, 21.6)			
Lithocholic acid						
Super-complier	3.30 (2.6, 4.1)	3.45 (2.8, 4.3)	3.73 (3.0, 4.7)	0.04 (0.2)	0.24 (0.2)	0.87
Goal-achiever	4.49 (3.7, 5.5)	4.57 (3.7, 5.6)	4.29 (3.5, 5.3)			
Taurochenodeoxycholic acid						
Super-complier	18.08 (15.1, 21.7)	15.64 (13.0, 18.8)	17.04 (14.2, 20.5)	-0.11 (0.2)	-0.09 (0.2)	0.99
Goal-achiever	21.26 (18.0, 25.2)	19.87 (16.8, 23.5)	21.33 (18.0, 25.3)			
Taurocholic acid						
Super-complier	5.31 (4.4, 6.4)	5.21 (4.3, 6.3)	5.48 (4.5, 6.7)	0.03 (0.2)	-0.03 (0.2)	0.99
Goal-achiever	6.49 (5.4, 7.8)	6.23 (5.2, 7.5)	6.85 (5.7, 8.2)			
Taurodeoxycholic acid						
Super-complier	6.23 (4.7, 8.2)	6.65 (5.0, 8.8)	6.77 (5.1, 8.9)	0.36 (0.3)	0.24 (0.3)	0.87
Goal-achiever	8.37 (6.5, 10.8)	6.97 (5.4, 9.0)	7.70 (6.0, 9.9)			
Ursodeoxycholic acid						
Super-complier	7.59 (5.3, 11.0)	5.87 (4.1, 8.5)	6.31 (4.4, 9.1)	-0.56 (0.4)	-0.50 (0.4)	0.87
Goal-achiever	4.11 (2.9, 5.8)	4.70 (3.3, 6.6)	4.84 (3.4, 6.8)			

[a] Means and 95% CIs are least squared means from linear mixed effects models, with a random effect for subject and an interaction term for visit*intervention arm.

[b] Beta coefficients and standard errors are from linear mixed effects models, with a random effect for subject and an interaction term for visit*intervention arm.

[c] P-values were estimated using likelihood ratio tests and were adjusted for multiple testing using False-Discovery Rate correction using the Benjamin-Hochberg method

Abbreviations: CI, confidence intervals; SE, standard error

Supplemental Table 5. Effects of strict adherence to the high-fiber, high-fruit and-vegetable, and low-fat diet on circulating total bile acids concentrations by baseline participant characteristics in the Polyp Prevention Trial, 1991-1998 (N=170 super-compliers and N=198 goal-achieving controls)

	Baseline	Year-2	Year-3	Intervention effect, Year-2	Intervention effect, Year-3		
	**Mean (95% CI)[a]**	**Mean (95% CI)[a]**	**Mean (95% CI)[a]**	**Beta Coef. (SE)[b]**	**Beta Coef. (SE)[b]**	***P*[c]**	***P-interaction***
Age							0.14
> 62							
Super-complier	730 (589.8, 902.9)	553 (447.1, 684.4)	618 (499.8, 765.2)	-0.31 (0.2)	-0.17 (0.2)	0.93	
Goal-achiever	792 (674.2, 930.9)	742 (631.5, 871.9)	758 (644.8, 890.3)				
< 62							
Super-complier	673 (573.4, 789.3)	721 (614.3, 845.6)	740 (631.0, 868.6)	-0.11 (0.2)	-0.10 (0.2)	0.99	
Goal-achiever	686 (573.7, 820.5)	794 (663.9, 949.4)	809 (676.6, 967.6)				
Sex							0.90
Male							
Super-complier	729 (624.1, 852.3)	716 (612.3, 836.2)	731 (625.4, 854.1)	-0.01 (0.2)	-0.07 (0.2)	0.99	
Goal-achiever	827 (713.5, 958.3)	819 (706.8, 949.3)	869 (750.2, 1007.4)				
Female							
Super-complier	644 (516.7, 802.2)	561 (450.6, 699.6)	632 (507.6, 788.1)	-0.30 (0.2)	-0.07 (0.2)	0.93	
Goal-achiever	647 (530.6, 787.8)	692 (568.2, 843.6)	669 (548.9, 815.0)				
Body mass index							0.52
Normal weight (18.5-24.99 kg/m^2)							
Super-complier	681 (467.6, 991.9)	684 (469.9, 996.7)	590 (405.3, 859.8)	0.19 (0.4)	-0.27 (0.4)	0.93	
Goal-achiever	637 (471.7, 859.4)	561 (415.5, 757.1)	667 (494.4, 900.7)				
Overweight (25-29.99 kg/m^2)							
Super-complier	927 (457.1, 1879.3)	620 (305.9, 1257.4)	714 (352.0, 1447.3)	-0.58 (0.6)	-0.61 (0.6)	0.93	
Goal-achiever	834 (514.6, 1351.6)	835 (515.5, 1354.1)	978 (603.3, 1584.6)				
Obese (> 30 kg/m^2)							
Super-complier	686 (532.7, 883.0)	633 (491.8, 815.1)	698 (541.9, 898.2)	-0.22 (0.2)	-0.13 (0.2)	0.93	
Goal-achiever	772 (609.4, 977.6)	831 (656.0, 1052.4)	859 (678.2, 1087.9)				
Smoking status							0.50
Smoker							
Super-complier	705 (593.4, 836.5)	602 (507.2, 714.9)	676 (569.6, 802.9)	-0.24 (0.2)	-0.07 (0.2)	0.93	
Goal-achiever	757 (645.7, 888.3)	765 (652.2, 897.3)	764 (651.2, 896.0)				
Non-smoker							
Super-complier	683 (562.4, 830.2)	708 (582.5, 859.9)	706 (581.2, 858.0)	-0.00 (0.2)	-0.07 (0.2)	0.99	
Goal-achiever	734 (612.9, 878.2)	762 (636.3, 911.6)	795 (664.6, 952.2)				
Baseline adenoma characteristics							0.44
Early adenoma							
Super-complier	740 (616.0, 889.5)	631 (525.1, 758.2)	739 (615.0, 888.0)	-0.15 (0.2)	0.04 (0.2)	0.93	
Goal-achiever	716 (603.8, 848.1)	675 (569.6, 800.1)	696 (587.0, 824.6)				
Advanced or multiple adenomas							
Super-complier	663 (555.2, 792.2)	665 (556.4, 793.9)	657 (550.4, 785.4)	-0.12 (0.2)	-0.15 (0.2)	0.93	
Goal-achiever	769 (651.7, 906.3)	834 (707.3, 983.7)	845 (716.5, 996.5)				
Baseline fiber intake							0.70
Fiber intake > 11.4 g/1,000 kcal/day							
Super-complier	614 (503.9, 747.8)	606 (497.3, 737.9)	610 (500.9, 743.3)	0.02 (0.2)	-0.06 (0.2)	0.99	
Goal-achiever	718 (607.2, 848.6)	699 (591.0, 826.0)	745 (630.2, 880.9)				
Fiber intake ≤ 11.4 g/1, 000 kcal/day							
Super-complier	769 (649.9, 909.7)	690 (583.0, 816.0)	765 (646.3, 904.7)	-0.28 (0.2)	-0.08 (0.2)	0.93	
Goal-achiever	780 (658.3, 924.9)	847 (714.7, 1004.1)	821 (692.3, 972.6)				
Baseline fat intake							0.54
Fat intake > 34.7 g/1,000 kcal/day							
Super-complier	730 (610.8, 871.9)	635 (531.8, 759.2)	706 (590.6, 843.1)	-0.24 (0.2)	-0.08 (0.2)	0.93	
Goal-achiever	705 (588.9, 844.0)	723 (603.7, 865.2)	723 (604.0, 865.5)				

Fat intake ≤ 34.7 g/1,000 kcal/day							
Super-complier	654 (541.9, 789.0)	669 (554.2, 807.0)	672 (557.2, 811.3)	0.00 (0.2)	-0.04 (0.2)	0.99	
Goal-achiever	783 (667.2, 917.7)	800 (681.9, 937.9)	830 (707.5, 973.1)				
Baseline fruit-vegetable intake							0.74
Fruits-vegetable intake > 2.8 svgs./1,000 kcal/day							
Super-complier	651 (535.5, 792.5)	640 (525.9, 778.3)	675 (555.1, 821.6)	0.00 (0.2)	-0.02 (0.2)	0.99	
Goal-achiever	732 (615.5, 871.5)	719 (604.0, 855.2)	771 (648.2, 917.8)				
Fruits-vegetable intake ≤ 2.8 svgs./1,000 kcal/day							
Super-complier	736 (621.1, 871.8)	660 (557.2, 782.1)	704 (594.5, 834.5)	-0.26 (0.2)	-0.11 (0.2)	0.93	
Goal-achiever	761 (645.6, 896.4)	815 (691.6, 960.2)	787 (668.0, 927.5)				
Intervention fiber goals met							0.81
Fiber goals met							
Super-complier	675 (571.1, 798.2)	637 (539.0, 753.3)	690 (583.7, 815.8)	-0.11 (0.4)	0.35 (0.4)	0.93	
Goal-achiever	656 (392.3, 1095.8)	667 (399.4, 1115.5)	526 (314.6, 878.8)				
Fiber goals unmet							
Super-complier	734 (591.3, 910.9)	677 (545.5, 840.4)	691 (556.7, 857.6)	-0.15 (0.2)	-0.17 (0.2)	0.93	
Goal-achiever	752 (667.3, 847.7)	770 (683.2, 867.8)	799 (708.8, 900.4)				
Intervention fat goals met							0.27
Fat goals met							
Super-complier	670 (581.5, 771.0)	598 (519.7, 689.1)	700 (607.7, 805.8)	-0.05 (0.3)	0.54 (0.3)	0.93	
Goal-achiever	1034 (740.1, 1444.9)	958 (685.7, 1338.7)	745 (533.4, 1041.4)				
Fat goals unmet							
Super-complier	723 (591.4, 884.6)	714 (583.9, 873.3)	680 (556.0, 831.6)	-0.06 (0.2)	-0.20 (0.2)	0.93	
Goal-achiever	725 (634.2, 828.2)	748 (654.8, 855.1)	782 (684.2, 893.5)				
Intervention fruit-vegetable goals met							0.29
Fruits-vegetables goals met							
Super-complier	709 (616.5, 816.3)	658 (571.9, 757.2)	741 (643.8, 852.4)	-0.07 (0.2)	-0.00 (0.2)	0.99	
Goal-achiever	729 (552.7, 961.7)	710 (537.9, 935.9)	763 (578.3, 1006.2)				
Fruits-vegetables goals unmet							
Super-complier	617 (443.9, 856.5)	609 (438.6, 846.1)	467 (336.4, 649.0)	-0.07 (0.2)	-0.46 (0.2)	0.93	
Goal-achiever	750 (657.1, 855.6)	776 (680.3, 885.9)	783 (685.8, 893.1)				

[a] Means and 95% CIs are least squared means from linear mixed effects models, with a random effect for subject and an interaction term for visit*intervention arm.

[b] Beta coefficients and standard errors are from linear mixed effects models, with a random effect for subject and an interaction term for visit*intervention arm.

[c] P-values were estimated using likelihood ratio tests and were adjusted for multiple testing using false-discovery rate correction using the Benjamin-Hochberg method

Abbreviations: CI, confidence intervals; SE, standard error

Supplemental Table 6. Associations[a] of bile acid concentrations with rectal tissue microbiome relative abundances at baseline (N=65)

	Relative abundance of *a priori*-selected bacteria[b]									
	Bacteroides	***Fusobacterium***	***Porphyromonas***	***Parvimonas***	***Peptostreptococcus***	***Gemella***	***Prevotella***	***Solobacterium***	***Dialister***	**Clostridiales**
**Bile acid, ng/mL**	**Rs; *P***	**Rs; *P***	**Rs; *P***	**Rs; *P***	**Rs; *P***	**Rs; *P***	**Rs; *P***	**Rs; *P***	**Rs; *P***	**Rs; *P***
Chenodeoxycholic acid	0.04; 0.74	0.09; 0.47	-0.11; 0.39	0.08; 0.54	0.08; 0.53	0.06; 0.63	-0.04; 0.76	0.18; 0.16	-0.19; 0.13	-0.25; 0.05
Cholic acid	-0.01; 0.95	0.01; 0.96	-0.04; 0.77	0.06; 0.62	0.06; 0.67	0.04; 0.78	0.05; 0.72	0.21; 0.10	-0.20; 0.11	-0.11; 0.40
Deoxycholic acid	0.11; 0.41	0.14; 0.27	-0.13; 0.31	-0.02; 0.89	-0.07; 0.61	0.002; 0.99	-0.22; 0.09	-0.02; 0.88	-0.12; 0.34	-0.21; 0.09
Glycochenodeoxycholic acid	0.21; 0.10	-0.03; 0.81	-0.08; 0.52	0.005; 0.97	-0.05; 0.69	-0.001; 0.99	0.04; 0.78	-0.05; 0.71	0.03; 0.84	-0.10; 0.43
Glycocholic acid	0.22; 0.08	-0.06; 0.65	-0.09; 0.48	-0.05; 0.69	-0.11; 0.38	-0.05; 0.68	0.06; 0.66	-0.13; 0.32	0.08; 0.53	-0.16; 0.20
Glycodeoxycholic acid	0.20; 0.12	0.03; 0.84	-0.15; 0.25	-0.10; 0.45	-0.18; 0.17	-0.07; 0.59	-0.11; 0.38	-0.19; 0.13	0.02; 0.88	-0.20; 0.11
Glycolithocholic acid	-0.01; 0.91	-0.18; 0.16	-0.18; 0.16	-0.07; 0.57	-0.11; 0.40	-0.07; 0.60	-0.11; 0.40	0.05; 0.72	-0.07; 0.61	0.10; 0.42
Glycoursodeoxycholic acid	0.16; 0.22	-0.05; 0.70	-0.15; 0.26	-0.11; 0.41	-0.12; 0.36	-0.10; 0.42	-0.09; 0.49	-0.18; 0.16	0.04; 0.77	-0.12; 0.35
Lithocholic acid	-0.22; 0.09	0.11; 0.39	0.04; 0.76	0.11; 0.38	0.09; 0.47	0.14; 0.28	-0.09; 0.50	0.18; 0.15	-0.13; 0.29	-0.05; 0.70
Taurochenodeoxycholic acid	0.30; 0.02	-0.10; 0.46	-0.07; 0.61	-0.03; 0.81	-0.05; 0.71	-0.03; 0.82	0.07; 0.59	-0.05; 0.68	0.10; 0.43	-0.20; 0.11
Taurocholic acid	0.27; 0.03	-0.03; 0.84	-0.01; 0.95	0.05; 0.72	0.04; 0.76	0.04; 0.77	0.15; 0.24	0.02; 0.89	0.12; 0.34	-0.23; 0.07
Taurodeoxycholic acid	0.28; 0.02	-0.01; 0.93	-0.12; 0.36	-0.11; 0.39	-0.15; 0.24	-0.08; 0.53	-0.02; 0.87	-0.17; 0.19	0.12; 0.35	-0.26; 0.04
Ursodeoxycholic acid	0.07; 0.58	0.09; 0.48	-0.12; 0.33	-0.04; 0.73	-0.03; 0.80	-0.05; 0.71	-0.13; 0.29	-0.02; 0.86	-0.12; 0.36	-0.20; 0.11
Summary scores										
Total bile acids	0.21; 0.10	-0.02; 0.90	-0.17; 0.17	-0.05; 0.67	-0.10; 0.42	-0.05; 0.68	-0.09; 0.50	0.01; 0.95	-0.10; 0.45	-0.20; 0.11
Primary bile acids[c]	0.02; 0.88	0.06; 0.67	-0.09; 0.47	0.07; 0.59	0.07; 0.59	0.05; 0.71	-0.01; 0.92	0.19; 0.13	-0.20; 0.12	-0.21; 0.09
Secondary bile acids[d]	0.11; 0.38	0.13; 0.33	-0.15; 0.24	-0.03; 0.83	-0.07; 0.58	-0.01; 0.95	-0.24; 0.06	-0.02; 0.87	-0.11; 0.38	-0.21; 0.10

[a] Spearman correlations were adjusted for age and sex.

[b] Bacterial relative abundance mean and standard deviation (SD): *Bacteroides* 15.88 (13.14), *Fusobacterium* 0.35 (0.78), *Porphyromonas* 0.19 (0.41), *Parvimonas* 0.02 (0.08), *Peptostreptococcus* 0.03 (0.09), *Gemella* 0.14 (0.57), *Prevotella* 0.36 (1.03), Solobacterium 0.01 (0.04), Dialister 0.39 (0.71), Clostridiales (order) 48.23 (18.85).

[c] Primary Bile Acids = log2 of the sum chenodeoxycholic acid, cholic acid, glycocholic acid, glycochenodeoxycholic acid, taurocholic acid, and taurochenodeoxycholic acid

[d] Secondary Bile Acids =log2 of the sum of deoxycholic acid, lithocholic acid, glycodeoxycholic acid, glycolithocholic acid, and taurodeoxycholic acid.
